# Effectiveness and economic impact of a diabetes education program among adults with type 2 diabetes in South Texas

**DOI:** 10.1186/s12889-021-11632-9

**Published:** 2021-09-09

**Authors:** Matthew Lee Smith, Lixian Zhong, Shinduk Lee, Samuel D. Towne, Marcia G. Ory

**Affiliations:** 1 Center for Population Health and Aging, Texas A&M University, College Station, TX 77843 USA; 2 Department of Environmental and Occupational Health, School of Public Health, Texas A&M University, College Station, TX 77843 USA; 3 College of Pharmacy, Texas A&M University, College Station, TX 77843 USA; 4 College of Nursing, University of Utah, Salt Lake City, UT 84112 USA; 5 School of Global Health Management and Informatics, University of Central Florida, Orlando, FL 32816 USA; 6 Disability, Aging, and Technology Cluster, University of Central Florida, Orlando, FL 32816 USA; 7 Southwest Rural Health Research Center, Texas A&M University, College Station, TX 77843 USA

**Keywords:** Diabetes education, Economic evaluation, Disease management, Community intervention

## Abstract

**Background:**

The long-term growth and sustained high prevalence of obesity in the US is likely to increase the burden of Type 2 diabetes. Hispanic individuals are particularly burdened by a larger share of diabetes than non-Hispanic White individuals. Given the existing health disparities facing this population, we aimed to examine the effectiveness and potential cost savings of the Diabetes Education Program (DEP) offered as part of Healthy South Texas, a state-legislated initiative to reduce health disparities in 27 counties in South Texas with a high proportion of Hispanic adults.

**Methods:**

DEP is an 8-h interactive workshop taught in English and Spanish. After the workshop, participants receive quarterly biometric screenings and continuing education with a health educator for one year. Data were analyzed from 3859 DEP participants with Type 2 diabetes living in South Texas at five time points (baseline, 3-months, 6-months, 9-months, 12-months). The primary outcome variable of interest for study analyses was A1c. A series of independent sample t-tests and linear mixed-model regression analyses were used to identify changes over time. Two methods were then applied to estimate healthcare costs savings associated with A1c reductions among participants.

**Results:**

The majority of participants were ages 45–64 years (58%), female (60%), Hispanic (66%), and had a high school education or less (75%). At baseline, the average hemoglobin A1c was 8.57%. The most substantial reductions in hemoglobin A1c were identified from baseline to 3-month follow-up (*P* < 0.001); however, the reduction in A1c remained significant from baseline to 12-month follow-up (P < 0.001). The healthcare cost savings associated with improved A1c for the program was estimated to be between $5.3 to $5.6 million over a two to three year period.

**Conclusion:**

Findings support the effectiveness of DEP with ongoing follow-up for sustained diabetes risk management. While such interventions foster clinical-community collaboration and can improve patient adherence to recommended lifestyle behaviors, opportunities exist to complement DEP with other resources and services to enhance program benefits. Policy makers and other key stakeholders can assess the lessons learned in this effort to tailor and expand similar initiatives to potentially at-risk populations.

**Trial registration:**

This community-based intervention is not considered a trial by ICMJE definitions, and has not be registered as such.

## Background

In 2018, more than 34 million Americans were estimated to have diabetes [1]. Further, there are an additional 88 million American adults estimated to have pre-diabetes in 2018 [1]. By 2060, prevalence of diagnosed Type 2 diabetes is expected to increase to 60.6 million among American adults [2]. This is driven, in part, by the rising rates of obesity, which is estimated to be approximately 42% [3]. Hispanic adults have higher rates of obesity and Type 2 diabetes than non-Hispanic White adults [4–6]. The likelihood of developing Type 2 diabetes among Hispanic individuals is 50% more than that of the average adult in the US [4]. These existing health inequalities make Hispanic individuals more susceptible to diabetes-related complications and an important population to receive diabetes management interventions.

People living with diabetes are at a greater risk of experiencing various diabetes-related complications (e.g., vision loss, nephropathy, and neuropathy) [7], lower quality of life [8, 9], and premature death [10, 11]. Healthcare expenditures for people with diabetes are almost two times higher than the healthcare expenditures for people without diabetes [12]. In 2017, the estimated economic cost of diabetes was $327 billion, which was substantially higher than the estimated cost of $245 billion in 2012 [13]. As such, diabetes in the US is a growing concern for individuals and their families as well as communities and other key stakeholders seeking to reduce the economic burden of diabetes throughout the US. Again, this underscores the urgent need to develop, deliver, and support diabetes interventions that can help susceptible and high-risk populations manage their diabetes.

Managing one’s blood glucose level is essential to diabetes care and self-management because it is associated with better health outcomes and reduced healthcare costs [13, 14]. An individual’s hemoglobin A1c value reflects their average blood glucose levels over approximately three months [15], and a higher A1c value indicates higher blood glucose level. A normal A1c level (measured in %) is below 5.7% [15], and American Diabetes Association (ADA) recommends A1c value below 7% for adults in general [4]. Unfortunately, about half of American adults with diagnosed diabetes have A1c value of 7.0% or higher [16]. Prior studies showed that reduction in A1c among people with diabetes can reduce risk of diabetic complications and healthcare cost [17, 18].

While interventions such as diabetes self-management education and support (DSMES) have been shown to improve diabetes self-management knowledge and skills [19, 20], they have also been successful to reduce A1c among participants [21–23]. Despite known benefits of DSMES, such programs are not always available or delivered in high-risk areas with disproportionate rates of obesity, pre-diabetes, and Type 2 diabetes [24, 25]. One such area with elevated diabetes risk is South Texas, a region of 27 counties near the Texas-Mexico border. In 2016, county-level prevalence of diabetes in the 27-county region ranged from 3.4 to 24.2% and 15 of the 27 counties had higher diabetes prevalence rate than the national prevalence rate of 10.2% [24, 25]. In this region, diabetes rates are among the highest in the US, which are largely attributed to the lack of healthcare resources needed [26]. As such, it important to deliver diabetes interventions in these underserved areas with high-risk populations as well as evaluate the effectiveness and impact such interventions have on individual and community health.

While DSMES has been offered in the South Texas region for the past two decades in the form of the Diabetes Education Program (DEP), its impact and cost effectiveness are not fully understood. Despite serving thousands of residents over time in this region, only limited evaluations of its impact have been performed [27]. In this context, the purposes of this study were to examine the changes in A1c and associated potential healthcare cost savings related to DEP implementation in South Texas among highly susceptible populations as part of the Healthy South Texas initiative.

## Methods

### Program description

The Healthy South Texas initiative was initiated in September of 2015 as a collaborative effort between government, academic, clinical, and community organizations to alleviate the impact of chronic and acute diseases throughout 27 South Texas counties [28]. Funded by the State of Texas on a biannual basis, this initiative integrated efforts between the Texas A&M Health Science Center and the Texas A&M Agrilife Extension System, and a primary focus was diabetes prevention and management. A hallmark program was the DEP, which was developed approximately 20 years ago by the Coastal Bend Health Education Center [29].

DEP is an ADA-recognized DSMES [29], which consists of an 8-h interactive workshop and four quarterly follow-up sessions (at 3, 6, 9, and 12 months). Available in either English or Spanish, the small group, 8-h interactive workshop was primarily delivered to participants during one full day; however, workshops are also commonly delivered as a series (e.g., two 4-h workshop sessions or four 2-h workshop sessions). One or more facilitators leads the workshop, and at least one facilitator was required to be a registered nurse, registered dietitian, pharmacist, or a certified diabetes educator. DEP was originally developed by a bilingual and bicultural team, along with community and clinical advisors, to ensure the program was culturally appropriate for target audiences. While the program was not specifically translated into Spanish, bilingual DEP facilitators led workshops for Spanish-speaking participants. Topics covered during DEP included diabetes and diabetes complications, blood glucose monitoring, diet, medication, physical activity, stress management and goal setting [27, 29]. Table 1 provides an overview of DEP in terms of topics of discussion and the amount of time spent on each topic. Each quarterly follow-up visit involved a brief one-on-one session between the participant and the DEP workshop leader to discuss the participant’s diabetes management goals and barriers and solutions to meet the goals. These follow-up sessions also included a brief data collection of A1c and other biometrics screenings.

**Table 1:** Overview of Diabetes Education Program agenda (delivered in one full day)

Time	Topic of Discussion
30 min	Data Collection:
	• Registration Forms
	• Pre-Test Instrument
	• Biometrics (e.g., A1c, blood pressure) (continues through the morning)
15 min	Introductions and Welcome
1.25 h	Diabetes Overview:
	• Preventing Short- and Long-Term Complications
15 min	Break
1.75 h	• Nutrition
	• Meal Planning
	• Basic Carbohydrate Counting
	• Label Reading
30 min	Working Lunch
45 min	Medications
30 min	Highs, Lows, and Sick Days
15 min	Introductions to Blood Glucose Meters & Monitoring (pre-exercise)
15 min	Physical Activity: “Walking Down Your Blood Sugar”
15 min	Monitoring (post-exercise)
15 min	Break
15 min	Foot Care
30 min	Life Stresses, Diabetes Stresses, Depression
30 min	Recommended Values
30 min	Goal Setting Activity
	Post-Test Instrument
	Course Evaluation

### Participants and recruitment procedures

Participants with pre-diabetes and diabetes were invited to participate in the program. Based on the community-based nature of this initiative, those without pre-diabetes or diabetes were also welcome to attend workshops; however, their data were not used for research purposes. Participants were recruited through recommendations and referrals from partnering healthcare systems and community-based organizations as well as through community health screening and educational events. Physical and electronic recruitment materials (e.g., flyers, social media) were also circulated, and self-referrals were accepted. Workshops were held in a variety of venues deemed to be convenient for participants to reduce burdens associated with transportation (e.g., healthcare settings, community centers, county offices) [30]. There was no charge to the participant for attending the DEP. The Coastal Bend Health Education Center consented participants to attend this community-based intervention and have their data used for evaluation purposes. Informed consent was obtained from all participants. Participant consent included information collected from participants during the intervention (i.e., registration forms, surveys, attendance, and biometric information), but did not include any information from their hospital records or claims data. This study involved retrospective reviews and data analyses and was reviewed and approved by the Institutional Review Board at Texas A&M University (IRB2019-0225D).

### Geospatial target area

Participants resided in multiple counties from the South Texas region; however, the majority lived within three counties. Over half of the participants resided in Nueces County, of which 63% were Hispanic and 11% had diabetes in 2018 [31]. The next largest segment of participants resided in Victoria County and Kleberg County, where the Hispanic population accounts for approximately 46 and 73% respectively, with a diabetes prevalence of 11 and 9% respectively in 2018 [31]. Given about 39% of all Texans are Hispanic and approximately 10% had diabetes statewide in 2018, this geographic area may carry disproportionate diabetes-related burdens and elevated risk for related complications. In light of this information, targeting residents of the South Texas region with a program addressing Type 2 diabetes was particularly relevant and timely.

### Measures

The primary outcome of interest was hemoglobin A1c levels (measured in % units). The health assessments were performed at baseline and each follow-up at 3, 6, 9, and 12 months. Participant characteristics were recorded in the participant registration form and included age (18–44, 45–64 and 65+), sex (male and female), race/ethnicity (non-Hispanic White, non-Hispanic Black, Hispanic, and other or multiple races), education (no high school, high school/GED, Associate’s degree, Bachelor’s degree, and graduate degree), insurance status (private insurance, Medicaid, Medicare, other insurance, and no insurance), smoking status (yes or no), and alcohol consumption status (yes or no). Diabetes characteristics (e.g., type) were also self-reported during the assessment.

### Statistical analysis

Baseline characteristics of program participants with Type 2 diabetes were described. Participant characteristics (categorical variables) were compared using Chi-squared tests based on whether they attended follow-up sessions at 3 months and 12 months. Post-workshop A1c changes at 3-month, 6-month, 9-month, and 12-month follow-up were compared to baseline using paired 2-sample t-tests. Linear mixed models with participant-level random intercepts were fitted for continuous outcome variables (A1c) controlling for covariates including age, sex, race/ethnicity, education, insurance status, and health behaviors (smoking and alcohol consumption). Linear mixed regression models were conducted using Stata SE14 (Stata Corporation, College Station, TX) using the “mixed” command. Statistical significance for all analyses was determined using the criterion of *p* < 0.05.

### Economic evaluation

Two methods were applied to estimate healthcare costs savings associated with A1c reductions observed from this initiative. Method 1 adopted Bansal and colleagues [32] approach and estimated healthcare cost savings associated with A1c reduction among participants with a baseline A1c ≥ 9%. In this study, Bansal and colleagues [32] utilized a large US health plan administrative claims database to analyze cost savings associated with A1c reduction over a 2-year period. The initial data source included more than 6 million members from the Optum Clinformatics Data Mart database with linked lab values (OptumInsight, Eden Prairie, MN, USA). From these data, 3197 eligible diabetes patients with initial A1c ≥ 9% were identified and categorized into two groups: decreasers (i.e., whose A1c decreased by any amount during the 1-year post-education period) and non-decreasers (i.e., whose A1c increased or did not change). Healthcare costs were compared between a matched sample of 912 decreasers and 912 non-decreasers based on 2014 US Dollars. The study found that patients in the decreaser group averaged a 24% ($2503) reduction of healthcare costs in the first year of follow-up and 17% ($1690) reduction in the second year. The cost categories included inpatient, outpatient, emergency department, pharmacy, and other costs. These per-patient cost savings were adopted in our study and were multiplied by the number of eligible patients to estimate the cost savings associated with the DEP over a two-year time period. Two types of cost savings were estimated: 1) cost savings from followed participants at 12 months; and 2) cost-savings extrapolated to the entire program participants, including those lost to follow-up. The cost-savings from followed participants at 12 months were directly computed by multiplying the per-patient cost savings by the number of participants with baseline A1c ≥ 9% and A1c reduction at 12-month follow-up. To estimate the cost savings for the entire program, we first calculated the proportion of Type 2 diabetes participants with reduction of A1c (decreasers) based on 12-month follow-up data among participants with baseline A1C ≥ 9%. This proportion was then applied to the program participants with baseline A1C ≥ 9% to estimate the number of eligible participants for cost savings. Although DEP data were only collected over a 12-month period, we estimated the potential 1-year and 2-year healthcare cost savings for this sample based on assumptions in savings similar to what Bansal and colleagues reported [31].

Method 2 was based on Glimer and colleagues [33], which assessed the impact of A1c change on healthcare costs among patients with diabetes. The study prospectively followed 1694 diabetes patients via a patient survey, which were then merged with a medical record review. Multivariate generalized linear regression analysis was conducted to predict cost differentials for 1% changes in A1c over a 3-year period. Costs were originally reported in 2002 US Dollars. The study found increasing cost differentials associated with 1% change of A1c as A1c levels increased. The study reported an average per patient cost saving of $1374 for A1c change from 10 to 9%, $1303 from 9 to 8%, $373 from 8 to 7%, and -$514 from 7 to 6% in the overall diabetes patient population. In our study, we evaluated the 12-month follow-up A1c change from baseline for each participant and segmented the change into 1% intervals (A1c < 7, 7% ≤ A1c < 8, 8% ≤ A1c < 9, 9% ≤ A1c < 10%, A1c ≥ 10%). Cost savings for that particular participant were estimated to be a linear combination of cost savings in each segment. For example, if a participant had a baseline A1c of 9.5% and a 12-month follow-up A1c of 7.5%, the participant would be assigned a cost-saving of ($373)*.5+ ($1303)*1 + ($1374)*0.5 = $2176.50.

Because Glimer and colleagues [33] capped cost savings at A1c levels of 10%, and our data reported A1c reductions in the ≥10% range, we extrapolated the cost savings by carrying forward the $1374 per 1% reduction to participants with to A1c ≥10%. For example, if a participant had a baseline A1c of 12%, and a 12-month follow-up A1C of 8.5%, they would be assigned a cost-saving of ($1374)* [2] + ($1374)*1 + ($1303)*(0.5) = $4773.5. The impact of A1c change on healthcare costs in both directions were considered. If a participant had an A1c increase at 12-month follow-up compared to baseline, then the sign would be reversed to reflect the cost differential. After estimating all participant’s cost savings based on their 12-month follow-up, average cost-savings were calculated based on baseline A1c distribution in the following categories (A1c < 7, 7% ≤ A1c < 8, 8% ≤ A1c < 9, 9% ≤ A1c < 10%, and A1c ≥ 10%).

For Method 2, two of cost savings were estimated: 1) cost-savings from followed participants at 12-month follow-up; and 2) cost savings extrapolated to the entire program participants including those lost to follow-up. To estimate the potential cost savings for the entire program, the proportion of participants’ baseline A1c distribution based on these A1c intervals were computed and applied to the average cost savings to generate a weighted average cost saving per participant. This weighted average cost saving per participant was then multiplied by the number of Type 2 diabetes patients who enrolled in the program at baseline.

For both Method 1 and Method 2 used in this study, all costs savings were inflated to 2018 US Dollars based on consumer price index (CPI) for medical care services. Discounting was not applied given the relative short time horizon of the program. Costs in 2018 US Dollars was used based on the most recent year of DEP data collected for these analyses.

## Results

### Sample follow-up and characteristics

**Fig. 1:**
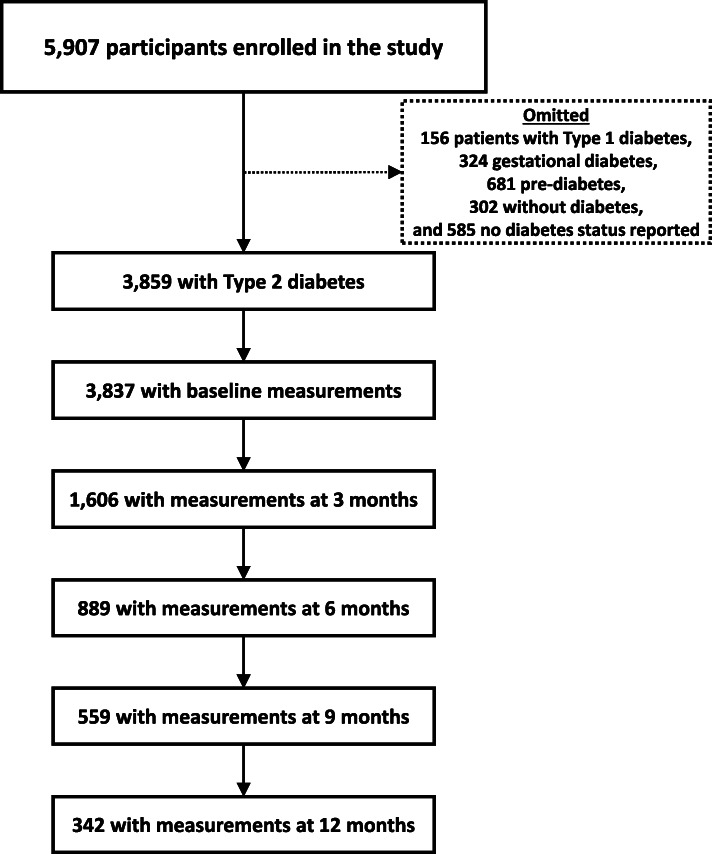
Flow chart of participants in the Diabetes Education Program from baseline to 12-months

Between September 1, 2015 and July 31, 2018, a total of 5907 participants enrolled in DEP. The majority (*n* = 3859) had Type 2 diabetes and 681 had pre-diabetes at baseline. The participants flow from baseline to 3-, 6-, 9-, and 12-month follow-ups are presented in Fig. 1. Table 2 presents the characteristics of these patients at baseline, 3-month follow-up, and 12-month follow-up. At baseline, most program participants were between the ages of 45 and 64 (58%), female (60%), Hispanic (66%), and had a high school education or less (75%). Most participants (81%) had insurance coverage, with 49% being covered by private insurance. Large proportions of participants did not smoke (89%) and did not consume alcohol (74%). The baseline average A1c level was 8.52% (±2.26%).

**Table 2:** Characteristics of participants at baseline, 3 months, and 12 months

	Baseline	3-mo assessment	no 3-mo assessment	P	12-mo assessment	no 12-mo assessment	P
	(***n*** = 3837)	(***n*** = 1606)	(***n*** = 2231)		(***n*** = 342)	(***n*** = 3495)	
	n ()	n ()	n (%)		n (%)	n (%)	
**Sex**				0.339			0.612
Male	1546 (40.35)	660 (41.25)	886 (39.71)		142 (41.64)	1404 (40.23)	
Female	2285 (59.65)	940 (58.75)	1345 (60.29)		199 (58.36)	2086 (59.77)	
**Race/Ethnicity**				< 0.001			< 0.001
Non-Hispanic Black	131 (3.43)	47 (2.94)	84 (3.79)		10 (2.92)	121 (3.48)	
Hispanic	2513 (65.80)	984 (61.46)	1529 (68.94)		175 (51.17)	2338 (67.24)	
Non-Hispanic White	1057 (27.68)	519 (32.42)	538 (24.26)		144 (42.11)	913 (26.26)	
Other/Multiple Races	118 (3.09)	51 (3.19)	67 (3.02)		13 (3.80)	105 (3.02)	
**Age**				< 0.001			< 0.001
18–44	558 (14.54)	187 (11.64)	371 (16.63)		30 (8.77)	528 (15.11)	
45–64	2209 (57.57)	931 (57.97)	1278 (57.28)		174 (50.88)	2035 (58.23)	
65+	1070 (27.89)	488 (30.39)	682 (26.09)		138 (40.35)	932 (26.67)	
mean (std. dev.)	57.10 (±12.23)	58.32 (±11.63)	56.22 (±12.57)		60.76 (±11.39)	56.74 (±12.25)	
**Education**				< 0.001			0.003
No High School (HS)	513 (16.99)	179 (14.49)	334 (18.71)		31 (13.14)	482 (17.31)	
HS/GED	1760 (58.28)	697 (56.44)	1063 (59.55)		123 (52.12)	1637 (58.80)	
Associate	321 (10.63)	155 (12.55)	166 (9.30)		38 (16.10)	283 (10.17)	
Bachelors	250 (8.28)	121 (9.80)	129 (7.23)		29 (12.29)	221 (7.94)	
Graduate	176 (5.83)	83 (6.72)	93 (5.21)		15 (6.36)	161 (5.78)	
**Insurance**				< 0.001			< 0.001
Private	1868 (48.80)	872 (54.43)	996 (44.74)		215 (63.24)	1653 (47.39)	
Medicaid	109 (2.85)	34 (2.12)	75 (3.37)		2 (0.59)	107 (3.07)	
Medicare	754 (19.70)	300 (18.73)	454 (20.40)		72 (21.18)	682 (19.55)	
Other	317 (8.28)	84 (5.24)	233 (10.47)		5 (1.47)	312 (8.94)	
No insurance	780 (20.38)	312 (19.48)	468 (21.02)		46 (13.53)	734 (21.04)	
**Smoke**				0.005			0.000
Yes	402 (10.53)	142 (8.89)	260 (11.70)		14 (4.12)	392 (11.20)	
No	3417 (89.47)	1455 (91.11)	1962 (88.30)		326 (95.88)	3109 (88.80)	
**Drink Alcohol**				0.027			0.762
Yes	1002 (26.35)	449 (28.22)	553 (25.01)		87 (25.66)	915 (26.42)	
No	2800 (73.65)	1142 (71.78)	1658 (74.99)		252 (74.34)	2548 (73.58)	
**A1c** (mean (std. dev.))	8.52 (±2.26)	7.41 (±1.69)	n/a		7.309 (±1.59)	n/a	

*n/a* not applicable

### Unadjusted A1c changes

**Fig. 2:**
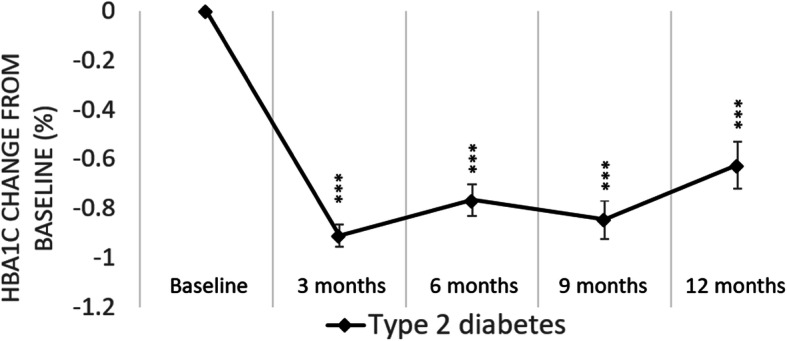
Unadjusted A1c change from baseline at 3, 6, 9, and 12 months. (Statistically significant compared to baseline ***P ≤ 0.001 **P ≤ 0.01 *P ≤ 0.05. Error bars indicate the standard error)

A1c changes from baseline at 3-, 6-, 9-, and 12-month follow-up are presented in Fig. 2. Participants showed statistically significant A1c reductions across all follow-up time points. On average, participants had a 0.90% point reduction of A1c at 3 months (*p* < 0.001), followed by 0.77% point reduction at 6 months (*p* < 0.001), 0.84% point at 9 months (*p* < 0.001), and 0.62% points at 12 months (*p* < 0.001).

### Adjusted A1c changes

Table 3 reports the A1c improvements between baseline and follow-ups after adjusting for covariates (i.e., age, sex, race/ethnicity, education, insurance type, smoking status, and alcohol consumption) using a linear mixed regression model. Statistically significant adjusted changes were detected. The adjusted A1c reductions were 0.93% points at 3 months (*p* < 0.001), 0.83% points at 6 months (*p* < 0.001), 0.87% points at 9 months (*p* < 0.001), and 0.73% points at 12 months (*p* < 0.001).

**Table 3:** Adjusted^a^ changes between baseline and follow-up means for improved health Outcomes

	From Baseline to 3-mo^b^			From Baseline to 6-mo^b^			From Baseline to 9-mo^b^			From Baseline to 12-mo^b^		
	Adjusted Change^a^	Std. Err.	P	Adjusted Change^a^	Std. Err.	P	Adjusted Change^a^	Std. Err.	P	Adjusted Change^a^	Std. Err.	P
**Type 2 diabetes**												
Average A1c	−0.926	0.045	< 0.001	−0.832	0.058	< 0.001	−0.870	0.070	< 0.001	−0.731	0.091	< 0.001

^a^ All changes were adjusted for age, sex, race/ethnicity, education, language, insurance type, smoking, and drinking status

^b^ Adjusted changes between baseline and 3 months, 6 months, 9 months, and 12 months were from linear mixed regression models

### Economic impact evaluation

Table 4 reports the estimated healthcare cost savings from A1c reductions for the first and second year following DEP participation using the Bansal et al. [31] approach. The cost savings per patient were $2780 in the first year and $1877 in the second year among participants with baseline A1c of 9% and above. Of the 3859 Type 2 diabetes participants who enrolled in DEP, 1375 (35.63%) had baseline A1c ≥ 9%, 1152 (83.75%) of which were estimated to have decreased A1c at 12-month follow-up. The cost savings of the T2DM participant based on 12-month follow-up was estimated to be $186 K in the first year and $126 K in the second year. When extrapolated to the total number of qualified program participants, the total cost savings of DEP were estimated to be $3.2 million in the first year and an additional $2.2 million in the second year.

**Table 4:** Estimated direct medical cost savings from the program using the Bansal approach

**Number of Type 2 diabetes participants at 12-month**^a^			**Number of Type 2 Diabetes participants at baseline**			
**- with baseline A1c > =9%**		80	**- with baseline A1c > =9%**			1375
**- A1c decreased at 12-month**		67	**- A1c decreased at 12-month (extrapolated)**			1152
**Estimated cost savings from followed participants (12 month) in 2 years post training**						
**Direct medical cost savings**	1st year (per patient)^b^	2nd year (per patient)^b^	1st year post DEP (based on 12-month follow-up)^c^	2nd year post DEP (based on 12-month follow-up)^c^	1st year post DEP (estimated total program saving)^d^	2nd year post DEP (estimated total program saving)^d^
**Medical**	2123	1236	142,241	82,812	2,445,696	1,423,872
**Inpatient**	510	476	34,170	31,892	587,520	548,352
**Outpatient**	1597	743	106,999	49,781	1,839,744	855,936
**-ER**	17	16	1139	1072	19,584	18,432
**Pharmacy**	288	394	19,296	26,398	331,776	453,888
**Others**	92	60	6164	4020	105,984	69,120
**Total (2014 USD)**	2503	1690	167,701	113,230	2,883,456	1,946,880
**Total (2018 USD)**	2780	1877	186,247	125,752	3,202,560	2,162,179

^a^ Estimates are from the program

^b^ individual cost savings are from Bansal 2018, cost saving only apply to patients with baseline A1c > =9% and decreased compared to the ones with baseline A1c > =9% and not decreased post index date

^c^ program cost savings were estimated by multiplying per patient per year cost by the number of qualified program participants at 12-month follow-up

^d^ program cost savings were estimated by multiplying per patient per year cost by the estimated number of total qualified program participants

Table 5 reports the estimated healthcare cost savings from A1c reductions in three years post DEP participation using the Glimer et al. [33] approach. The average cost savings per participant were estimated by initial A1c levels (i.e., < 7%, 7–8%, 8–9%, 9–10%, and ≥ 10%) based on 12-month follow-up data. The weighted average cost saving was $1501 per participant, and the total program healthcare cost saving of the 3859 Type 2 diabetes participants who enrolled in DEP was estimated to be $5.8 million for three years in 2018 US Dollars.

**Table 5:** Estimated direct medical cost savings from the program using the Gilmer approach

A1c change from baseline in Type 2 diabetes patients at 12 months						
Baseline A1c levels	A1c < 7%	7% ≤ A1c < 8%	8% ≤ A1c < 9%	9% ≤ A1c < 10%	A1c ≥ 10%	
No. of Type 2 diabetes participants with 12-mo follow-up	133	78	47	27	53	
Average A1c change at 12-month follow-up (std. dev.)	0.15(±0.73)	−0.24(±0.90)	−0.45(±1.68)	−1.37(±1.55)	−2.9(±2.56)	
Number of A1c decreasers	58	50	32	22	45	
Number of A1c increasers	64	24	14	5	8	
Number with no A1c change	11	4	1	0	0	
Cost savings for 1% reduction	−514	373	1303	1374	1374	
Average cost savings per person by initial A1c level	−58	− 220	−130	1057	3406	
Program participants initial A1c distribution	28.9%	20.5%	14.9%	11.6%	24.0%	
	2002 USD					2018 USD
average cost savings per person (weighted) program saving in 3 years based on 12 m follow-up Estimated current program total cost saving in 3 years	$859					$1501
	$178,073					**$311,299**
	$3,314,186					**$5,793,715**

## Discussion

This study presented changes in A1c among DEP participants and associated cost savings. After participating in DEP, participants showed an average reduction in their A1c after 12 months. This outcome is comparable to A1c reduction reported in other DSMES studies. For example, a review of DSMES interventions observed A1c reduction of 0.88 percentage points for an intervention that involves both group and individual engagement [17]. This rate is slightly higher than the observed A1c reduction of group-based diabetes education programs and slightly lower than the observed A1c reduction of diabetes self-management programs delivered in community settings [18, 22, 34–36].

The current study reports estimated healthcare cost savings associated with A1c reductions among DEP participants, as shown using two previously reported cost savings estimation methods [32, 33]. The total cost savings (i.e., $5.4 million for two years) extrapolated based on the Bansal’s estimation was higher than the amount of cost saving extrapolated based on the Gilmer’s estimation ($5.8 million for three years). The reason for this difference points to the Bansal estimation utilizing cost savings of patients with baseline A1c levels ≥9 percentage points [32] and the Gilmer estimation also including patients with baseline A1c levels ≤9 percentage points [33]. Further, the magnitude of A1c reduction was reflected Gilmer’s estimation, but not Bansal’s estimation. Because Bansal did not fully present the cost savings estimation by the magnitude of A1c reduction, the mean cost savings was used (i.e., $2503 for the first year and $1690 for the second year) in the cost savings calculation in the current study. Considering the mean A1c reduction of 2.3 percentage points among the decreasers in the Bansal’s study, and the mean A1c reduction of 0.73 to 0.93 percentage points in the current study, using the Bansal’s estimation may have slightly overestimated the cost savings of A1c reduction from the DEP.

The estimated yearly healthcare cost savings for this DEP was about $830 per participant (i.e., $2780× 1152 ÷ 3859) for the first year post DEP and $560 (i.e., $1877× 1152 ÷ 3859) for the 2nd year post DEP (i.e., ($2780 + $1877) ÷ 2 × 1152 ÷ 3859) using Bansal’s estimation. Using Glimer’s estimation, and healthcare cost saving per participant was estimated to be $1501 over three years post DEP. While we were not able to report yearly cost savings, this is roughly equivalent with an average $500 per person per year over three years (i.e., $1501÷ 3). Given that the cost savings probably peak in the first year and attenuates over time, the first year post DEP cost saving is likely to be more than $500 per person. These numbers are comparable to the estimated cost savings reported in the prior studies. For example, Turner and colleagues [35] examined 12-month pre- and post-education claims data for the Better Choices, Better Health diabetes program participants and found $815 direct cost savings through the program and $1504 indirect cost savings through the changes in related comorbid disease burden. Other studies that estimated cost savings of DSMES based on quality-adjusted life-years also showed potential economic benefits of DSMES [19, 37–39]. The current study estimated cost savings solely based on the A1c reduction; therefore, the estimated cost saving may underestimate the program impacts on the cost via enhanced quality of life and other comorbid disease burden. However, as stated by Turner and colleagues [35], not all DSMES are equal, and the economic benefits observed in this study may not be generalized to other programs or delivery settings. As such, further evaluation efforts are needed to generate more refined assessments of cost savings by population and delivery setting.

In the current study, the most dramatic A1c reduction occurred at 3-month follow-up, and the magnitude of A1c reduction decreased over time. While such tapering intervention effects can be common [40], this result may also be confounded by the potential bias associated with participant attrition and/or the effectiveness of the quarterly DEP follow-up sessions. For example, attrition may mitigate the tapering effects of an intervention because the healthiest participants may be most likely to remain over time. The high rates of participant attrition hold significant implications for DSMES in practice and research. In the current study, among those with Type 2 diabetes at baseline, 42% of participants had initial 3-month follow-up data and less than 10% had 12-month follow-up data. While the findings are encouraging for this DEP program, the findings should be interpreted in the context of a potential healthful bias related to program retention over time. One of the lessons learned from this intervention is that future efforts should emphasize the importance of program retention and aim to reduce barriers to follow-up and longer-term engagement with Type 2 diabetes participants. Tapering of intervention effects and attrition issues may speak to the overall DEP structure or delivery, which may call for refined follow-up session formats to keep participants engaged and optimize support for A1c self-management. While there are many possible reasons for attrition over a 12-month intervention (e.g., perceived value of the intervention, competing demands for participant time and resources, participant relocation), program coordinators should remain diligent to engage participants more regularly during the 3-months between data collection and strategically employ incentives and rewards to keep participants interested in the program. This study used its findings to extrapolate cost savings to the broader participant base who enrolled in the intervention; however, future efforts should attempt to maximize participant retention over longer durations for more accurate economic evaluations.

### Limitations

This study has several limitations. First, the high attrition rate was a significant finding, which prohibited obtaining A1c measures over time for all participants with baseline data and may have introduced bias. Attrition rates at 12-month follow-up were higher among younger participants, Hispanics, uninsured or Medicaid-insured, those with lower education, and those who engaged in smoking and alcohol consumption behaviors. While attrition is not unique to this study, especially given the relatively long timeline for follow-up, findings should be interpreted in light of this shortcoming. Second, the lack of a comparison group limited this study’s ability to examine the direct effects of the program rather than potential effects from other factors. Despite the limitation, the naturalistic study approach (i.e., uncontrolled by researchers) provides valuable insights that can be transferred to actual program delivery in the field. Third, the cost savings in this study were extrapolated based on two prior studies of cost savings from A1c reduction [32, 33]. The differences between the current study population and the participants of the previous two studies may have influenced the estimates and extrapolation of cost savings. For example, Bansal’s cost saving was estimated among those with a minimum of two years of commercial insurance or a Medicare Advantage plan during the study period. Bansal’s study showed greatest cost saving from outpatient care and limited cost savings from emergency care. In the current study, about 20% were uninsured, and this population might have different patterns and rates of health service utilization and resulting healthcare costs. Rather than relying upon previous studies for healthcare utilization and cost-related information, future studies should access the participants’ actual medical data (e.g., hospitalizations, emergency department visits, healthcare costs) to more accurately identify the effectiveness of interventions such as DEP. Finally, unlike cost-benefit studies that estimate both the cost of delivering the programs and cost saving from the program, this study did not estimate the cost of DEP delivery. Combined with higher attrition rates among younger participants, Hispanics, and less-resourced  individuals (e.g., uninsured or Medicaid), the estimated cost savings should be considered with caution for informing dissemination decisions.

## Conclusion

Findings support the benefits of DEP workshops with ongoing follow-up for sustained Type 2 diabetes risk management. In addition, study findings suggest significant potential cost savings as a result of improved blood glucose control among program participants. While this study is not without limitations, the findings indicate the potential of DEP and similar programs to reach more underserved and at-risk populations, such as the largely Hispanic population recruited in the current study. This study holds important implications for stakeholders seeking to ameliorate health disparities in diabetes and presents broader implications for potential cost savings for multiple public and private payers. Further work should be undertaken to investigate the program impacts based on direct measures of participant’s health service utilization and healthcare cost and compare the program effectiveness by population characteristics and delivery settings.

## Data Availability

Public access to the database used in this study is closed. The data that support the findings of this study are available from the Coastal Bend Health Education Center; however, restrictions apply to the availability of these data, which were used under agreements for the current study, and so are not publicly available. Data are, however, available from the authors upon reasonable request and with permission of the Coastal Bend Health Education Center.

## Abbreviations

DEP: Diabetes Education Program
US: United States
ADA: American Diabetes Association
DSMES: Diabetes Self-Management Education and Support
HS: High School
GED: General Education Development
